# Molecular Rationale of Insect-Microbes Symbiosis—From Insect Behaviour to Mechanism

**DOI:** 10.3390/microorganisms9122422

**Published:** 2021-11-24

**Authors:** Sujata Singh, Archana Singh, Varsha Baweja, Amit Roy, Amrita Chakraborty, Indrakant Kumar Singh

**Affiliations:** 1 Molecular Biology Research Lab, Department of Zoology, Deshbandhu College, University of Delhi, Kalkaji, New Delhi 110019, India; sujatasingh@db.du.ac.in (S.S.); vbaweja@db.du.ac.in (V.B.); 2 Department of Botany, Hansraj College, University of Delhi, New Delhi 110007, India; archanasingh@hrc.du.ac.in; 3 DBC ***i***4 Center, Deshbandhu College, University of Delhi, Kalkaji, New Delhi 110019, India; 4 EVA 4.0 Unit, Faculty of Forestry and Wood Sciences, Czech University of Life Sciences, Kamýcká 129, Suchdol, 16521 Prague 6, Czech Republic; roy@fld.czu.cz; 5 Excelentní Tým pro Mitigaci (ETM), Faculty of Forestry and Wood Sciences, Czech University of Life Sciences, Kamýcká 129, Suchdol, 16521 Prague 6, Czech Republic

**Keywords:** insect symbiosis, holobiont, gut microbiome, arthropod vector, host immunity, nutrition provisioning, detoxification, omics technology

## Abstract

Insects nurture a panoply of microbial populations that are often obligatory and exist mutually with their hosts. Symbionts not only impact their host fitness but also shape the trajectory of their phenotype. This co-constructed niche successfully evolved long in the past to mark advanced ecological specialization. The resident microbes regulate insect nutrition by controlling their host plant specialization and immunity. It enhances the host fitness and performance by detoxifying toxins secreted by the predators and abstains them. The profound effect of a microbial population on insect physiology and behaviour is exploited to understand the host–microbial system in diverse taxa. Emergent research of insect-associated microbes has revealed their potential to modulate insect brain functions and, ultimately, control their behaviours, including social interactions. The revelation of the gut microbiota–brain axis has now unravelled insects as a cost-effective potential model to study neurodegenerative disorders and behavioural dysfunctions in humans. This article reviewed our knowledge about the insect–microbial system, an exquisite network of interactions operating between insects and microbes, its mechanistic insight that holds intricate multi-organismal systems in harmony, and its future perspectives. The demystification of molecular networks governing insect–microbial symbiosis will reveal the perplexing behaviours of insects that could be utilized in managing insect pests.

## 1. Introduction

The American author Frederick Lenz beautifully said, “Symbiosis is a much higher reflection of intelligent life.” He termed a symbiotic relationship as reciprocity between two people governed by specific terms under certain conditions for a balanced relationship. It profoundly describes the complexity and diversity of the lifelong association of a microbial community with its eukaryotic hosts. The advancement in sequencing and PCR-based technologies has dramatically expanded our insight into microbial systems. Notwithstanding, the failure to cultivate these microbes in vitro impedes the dissection of the functional aspects of these interactions. This is primarily the reason behind the elusiveness of microbial consortiums [1,2]. Compared to higher eukaryotes, which harbour a panoply of commensal organisms, less-diverse microbial symbionts colonize insects. This enables investigators to focus on the contribution of individual symbionts to their host and project insects as an economical and excellent model system for interdisciplinary research [3,4,5]. The interdisciplinary research involving microbiology, neurosciences, and medicine holds a promising future for elucidating the role of gut symbionts in neurological and behavioural disorders (such as neurodegenerative disorders and autism spectrum disorders) and finding their probiotic solutions [6].

Furthermore, dysbiosis reduces the host fitness and inflicts autoimmune diseases such as rheumatoid arthritis or type I diabetes [7,8]. It is apparent that the host–microbe interaction is intricate, and any disturbance could have far-reaching impacts than hitherto appreciated. In insect–plant interactions, mutualistic symbiosis effects are considerably subtler [9]. Microbial mutualists often shape the diet breadth of insect hosts via nutrient provisioning and breaking down toxins, host adaptations to environmental perturbations, host behaviours, growth and development, mating, reproduction, plant physiology favouring insect hosts, and defending hosts against predators [10,11,12,13]. However, the gut bacteria have prioritised their role. Nutrient provisioning is the most crucial role, other than digestion and detoxification [14]. The symbiotic microbiome maintains and/or enhances the host immune system in insects serving as disease vectors. It also influences its vector competence [15]. As insects are prevalent human disease vectors and agricultural and forest pests, a better understanding of insect pests as a holobiont is of utmost importance for formulating sustainable management strategies (Table 1).

## 2. A Glimpse of Insect–Microbe Niche Foundations

### 2.1. Morphology and Physiochemical Conditions of Niches

Symbionts (i.e., viruses or archaea, bacteria, protist, and fungi) direct the insect’s growth and development trajectory. The basic niche foundation is established in conjuncture by both insects and symbionts [41,42]. This co-constructed niche is pivotal in insect diversification and is responsible for their eco-evolutionary success [43]. Besides multiple habitat options inside insects, the cuticles and gut are most accessible to microbial colonists. The ability of microbes to breach the exoskeleton and gut wall provides them access to hemocoel and insect cells [13]. Cuticles, a crucial physical barrier, can host more than thousands of bacterial cells. Cuticular invaginations (such as mycangia) and glandular invagination (like crypts or foveae) act as culture vessels; they protect microorganisms from abiotic factors and contamination [44,45]. However, some physical and chemical disturbances like ecdysis, antimicrobial secretions, and grooming behaviours limit microbial growth [46].

The attributes of the insect gut, such as the availability of nutrients and protection from desiccation and UV, make the gut favourable for colonisation. However, it also poses multiple challenges for microbes, such as (a) an unfavourable oxygen content, redox potential, and pH in the gut lumen; (b) digestive enzymes secretion; (c) physical disturbances like peristalsis; (d) secretions of the immune system; (e) habitat loss during insect moulting; and (f) competition among microbes for resources and colonisation sites. The hazard and resource accessibilities vary within the gut compartments with the insect stages and across insect groups based on their feeding ecology [10,47]. In most insects, the hindgut bears a more significant proportion of the microbial population. For example, in termites and scarab beetles, the hindgut acts as an anoxic fermentation chamber in which microbes degrade complex plant materials. In *Cortaritermes*, an integrative omics approach characterised carbohydrate-active enzymes from *Fibrobacteres* and *Spirochaetae*. They are present in the termite gut to overcome lignocellulose recalcitrance of the *Miscanthus* diet [48]. The spectra of the termite–fungal community were found stable across diverse host species from different habitats [49].

In comparison, an analysis of bacterial communities in termite guts and galleries demonstrated a unique pattern [50]. With the flux of digestive enzymes and immunologically active compounds, the midgut is hostile to microorganisms. Most insects have mild acidic to neutral pH (pH 6–7) in the midgut, favouring diverse microorganisms’ growth. However, some insect guts like lepidopteran have alkaline midguts (pH 8–12), inimical for microbial growth. Besides chemical barriers, the peritrophic matrix (PM) poses a physical barrier to microbial colonisation [13]. The majority of microorganisms fails to penetrate the PM and pass passively along with food [51,52]. Some bacterial communities reside in ectoperitrophic spaces. In some insects, the crop (food storage organ) is the primary habitat for microorganisms. However, regular food evacuation makes it a provisional habitat. In insect vectors, the foregut is the site of microbial adhesion for plant or animal pathogens.

Furthermore, certain insect groups have specialised cells, i.e., bacteriocytes (for bacteria) and mycetocytes (for yeast) for maintaining and hosting microorganisms. Microbes are restricted to these cells, with no access to the external environment, and are vertically transmitted [53]. The insects belonging to different feeding guilds with divergent evolutionary trajectories also shaped their microbial partners during evolution. Lepidopteran larvae feed on foliage and have simple gut morphologies that support a simple gut microbiome [54], including genera *Pseudomonas*, *Enterobacter*, *Enterococcus*, and *Klebsiella,* along with a few fungi. In contrast, the coleopteran gut is highly segmented, with certain modifications and enlargements based on their diet. They show marked variations in gut microbial communities [55,56]. In comparison to holometabolous insects, hemipterans gut tissues and microbiomes show significant modifications. The sap feeders lack PM and consume an extreme diet, harbouring symbionts primarily for nutrient provisioning [53].

### 2.2. Impact of Host Immune System on Resident Symbionts

**Figure 1:**
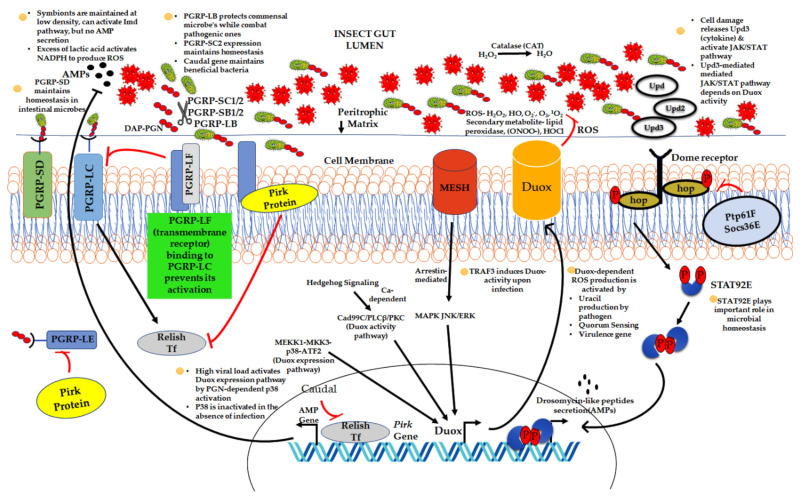
Depictions of different mechanisms involved in the maintenance of microbial homeostasis in the insect gut. The microbial homeostasis is attained by AMPs and reactive oxygen species (ROS) production. The Imd pathway is activated during high viral loads by recognising bacteria-derived peptidoglycan by the cell surface protein (PGRP-LC) and cytoplasmic receptor (PGRP-LE). PGRP-SD enhances Imd pathway signalling. AMPs are produced by the Imd pathway in the gut. Texts written in red are the negative regulators of the Imd pathway. Duox (member of NADPH oxidase family) produces ROS. It is mainly regulated by the Duox activity pathway and Duox expression pathway. Recently, MESH-regulated Duox activity has also been reported. Besides Imd pathway-mediated AMP production, the JAK/STAT pathway also produces a few AMPs. The combining of Upd molecules (cytokines) to the Dome receptor activates JAK/STAT signalling. Ptp61F and Socs36E are the negative regulators of the JAK/STAT pathway. The text written in green describes the regulatory aspect of different mechanisms in microbial homeostasis. DAP PGN—Diaminopimelic acid (DAP)-type peptidoglycan (PGN), PGRP—PGN recognition peptide, AMPs—antimicrobial peptides, Dome—domeless, Hop—hopscotch, Upd—unpaired, and TRAF3—Tumour necrosis factor receptor (TNFR)-associated factor 3. Pathogen—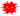. Beneficial or commensal bacteria 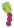.

Besides various factors (like the host diet, pH, pathogenic invasion, and ecological niche of the host) that affect the gut microbiome composition, there is firm evidence indicating the influence of the insect gut immunity in shaping the resident microbial community structure. Recently, an ant microbiome association suggested the importance of social interactions in shaping the host–microbiome [57]. Additionally, the composition of the microbiome varies across distinct nest chambers [58]. The painstaking efforts of insects to resist and eliminate foreign pathogens or opportunistic microbes while spurring beneficial microbes are crucial to insect survival and eco-evolutionary success (Figure 1). This perplexing immune response in the alimentary canal to eliminate invasive pathogens and simultaneously encourage or tolerate commensal microorganisms ensures homeostasis in the gut. For maintaining microbial homeostasis, different immune regulatory mechanisms have been deciphered. This includes (a) the immune deficiency (Imd) pathway, (b) dual oxidase-reactive oxygen species (Duox-ROS), (c) Janus kinase signal transducer, and (d) JAK/STAT pathway (activators of signal transduction) [59].

## 3. Microbial Symbiont: A Stealthy Modulator of Insect–Plant Interactions

**Figure 2:**
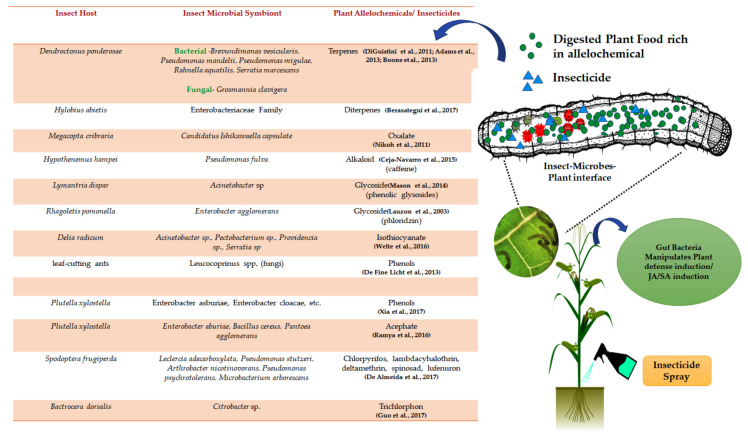
Pictorial representation of the insect–microbes–plant interface inside the gut of phytophagous insects. Insect-associated microbes manipulate host plant defence induction and metabolise/detoxify phytotoxins. Insect mutualists also detoxify insecticides and enhance the host fitness.

The ramification of insect–microbes symbiosis is considerably subtler in insect–plant interactions [60]. Insect mutualists allow insects to colonise diverse plant species by actively manipulating host plant physiology and enhancing the antiherbivore defence in their favour [61]. However, the extent to which symbionts incline the balance favouring host insects is still ambiguous and needs further experimentation. The role of microbial symbionts, “the hidden players”, is currently underappreciated. The herbivorous insects acquire gut microbial communities from their respective host plants. Hence, they vary with the host plant range [62]. Plant-derived allelochemicals often shape the gut microbiome by stimulating or inhibiting the growth of their respective microbial communities [63,64]. The microbial community structure also depends on the insect gut environment, microbial source, and plant genotype [65]. The gut microbiome plays a critical role in influencing the plant defence efficacy on phytophagous insects (Figure 2). The microbial symbiont (a) alters the efficacy of plant toxins targeting the insect gut peritrophic matrix (b) metabolises/detoxifies/degrades plant defensive secondary metabolites, and (c) modulates the induction of plant defence signalling [47,66,67].

The peritrophic matrix, a protective barrier of coleopteran and lepidopteran guts, is one of the critical targets of plant defensins (chitinase and protease). The disruption of the integrity of the PM by plant hydrolytic enzymes also co-opt resident gut bacteria for synergising their total activity [68]. It has been well-documented that some of the gut symbionts can effectively metabolise plant-derived toxic chemicals (phenols, terpenoids, alkaloid (caffeine), and glycoside) and render them inoperable [69,70,71]. The coevolutionary adaptation of *Acromyrmex echinatior*. (leaf-cutting ant) with *Leucocoprinus gongylophorus* (fungal symbiont) allowed the selection of the laccase enzyme in the fungal cultivar. It imparted a fitness advantage to ants by successfully detoxifying phenolic compounds using laccase and alleviating dietary challenges [72]. The isolation of gut microbial strains from *Delia radicum* (Cabbage root fly) identified the plasmid-carrying *saxA* gene among some isolated strains. The product of the *saxA* gene could degrade isothiocyanate, an insecticidal toxin of cabbage [73].

Similarly, metagenomics of the *Plutella xylostella* gut microbiota provided insight into the enrichment of genes involved in digestion, amino acid synthesis, and the detoxification of plant phenolics [74]. Finally, identifying core bacterial and fungal populations in the gut of bark beetles feeding on conifers paved the way for the improved knowledge of insect adaptation to conifer feeding as a holobiont [75,76]. The fungal symbiont, *Ceratocystis polonica* of bark beetles *(Ips typographus*), could effectively metabolise Stilbene, an antifungal compound in Norway spruce. It benefits bark beetles [77]. Recently, studies have been performed to comprehend the role of a microbial symbiont in metabolising insecticides (Figure 2). The promising role of gut bacteria-driven insecticide detoxification/degradation has been found [78,79]. For example, the gut-associated bacterial community of *Plutella xylostella* (a crucial pest of cruciferous) contains *Enterobacter aburiae*, *Bacillus cereus*, and *Pantoea agglomerans*, which aid in Acephate degradation [80]. Likewise, exploring the *Spodoptera frugiperda* gut microbial community documented an excellent reservoir for insecticide-degrading bacteria [81].

Besides degrading plant secondary metabolites, insect mutualists could modulate induced plant defences by quenching the free radical activity, utilising JA/SA antagonism, favouring the insect host and suppressing the expression of the plant defensive gene [82,83,84,85] (Figure 2). The evidence supports the interference in the insect-induced plastic phenotypic response in plants by microbial mutualists [19,23]. However, the impact of insect mutualists on the host fitness is obscure. The symbiotic partner might contribute a new genetic resource that gives its host the ability to synthesise bioactive molecules. For example, a crucial and well-studied elicitor (N-acylamino acids) of plant defences, widespread in the oral secretion of chewing insects, has been discovered to be synthesised in vitro by the gut symbiotic bacteria of noctuid caterpillars [86]. Another example from leaf miners involves the maintenance of the “green island” (photosynthetically active green area in senescent leaves) in host plant leaves. The leaf-mining moths (*Phyllonorycter blancardella*) harbour *Wolbachia* (a bacterial symbiont), which produces cytokinins, responsible for green island formation. The removal of *Wolbachia* leads to the disappearance of the green island and increased moth mortality [24]. Still, our understanding of the role of microbes as a mediator in insect–plant interactions is nascent. It requires comparative studies between related herbivore insects varying in diet breadths and manipulation of the gut microbial community. It is worth mentioning here that the manipulation of symbiotic association is challenging in many insect orders.

## 4. Microbiome Sabotaging the Vector Competence of Insect Hosts

Several human pathogens are circulated in the population by insect vectors, particularly mosquitoes. This has impacted human health globally. Intriguingly, most of these disease carriers have an innate resistance to the vectored pathogen. Different studies have demonstrated that only a tiny section of insect vectors has a thriving infection to transmit to healthy hosts successfully. However, most insects eliminate pathogens in the midgut soon after a bloodmeal, based on vector competence. Vector competence is the genetic ability of pathogen transmission by host insects. It is based on insects’ immune system proficiency that governs multiple immunity pathways [15,87]. To understand insect responses to pathogen infections, high-throughput gene profiling and reverse genetic analysis, i.e., the RNA interference (RNAi) approach, was used, which recorded the induction of a large set of innate immunity genes [88]. Furthermore, the rearing of insect vectors such as *Aedes aegypti* aseptically recorded a higher fold of pathogen infection compared to the wild-type. Such observations implicated the role of microbial fauna in modulating the immune resistance and vector competence of the host insect [89].

Here, we reviewed the influence of insect symbiotic microbiota on arboviral transmission and the intricacy of interactions modulating the vectorial capacity of arthropods, particularly vector competence. The core component of vector competence that we have highlighted is the proficiency of the host insect immune system and its responses to microbial challenges—how the microbial fauna of a vector modulates the transmission of arboviruses. The mechanisms underpinning the inherent symbiotic microbiota in arthropods to reduce arboviral transmission and pathogen blocking could be harnessed as a potential disease control of arthropod-borne diseases.

### 4.1. Arthropod Vector and its Symbiotic Microbiota 

Bacterial symbionts found in mosquitoes, sandflies, and ticks dominantly belong to Actinobacteria, Bacteroidetes, Firmicutes, and Proteobacteria [90,91,92,93]. They live in the gut and hemocoel. Symbionts have been reported from insects relying on a nutritionally deficient diet like vertebrate blood, plant phloem, and wood. Such symbiotic associations fulfil their nutrient needs in their diet. They offer mutualistic symbiosis and are known as primary symbionts. The quality of being indispensable for their hosts made them evolve to be vertically transmitted within their hosts. This ancient relationship shares a long coevolutionary history. It has made drastic changes in primary symbionts, such as reducing the genome size, gene loss, and selecting essential genes and pathways that favour unique niches in their host. For instance, *Buchnera aphidicola*, *Wigglesworthia*, and *Blochmannia* [94] are some of the more well-studied symbionts. *B. aphidicola* is an obligate endosymbiont of aphids. It lives distinctively inside host cells, i.e., bacteriocytes, and has customized its genes to provide aphids with essential nutrients and proteins deficient in their diet [32]. Likewise, *Wigglesworthia* is also an obligatory endosymbiont that resides in the bacteriome organ of tsetse flies. It is essential for their immune system development [33,95]. Another category of transient symbionts and originating recently in insects is commensal microbes known as secondary symbionts. Compared to the primary symbiont, secondary symbionts are dispensable, and their transfer from mother to progeny shows a lower fidelity. They can be transferred through different means, such as vertical, horizontal, or acquired from the environment—for example, *Hamiltonella defensa* from sap feeders and *Sodalis glossinidius* from tsetse flies. *Hamiltonella defensa* is a sporadic endosymbiont of sap-feeding insects that prevents the attack of parasitic wasps and protects them [96]. *Sodalis glossinidius*, an intracellular symbiont of the tsetse fly, lives in different tissues, including the gut lumen [97]. Besides mutualistic and commensal microbes, most insects also carry parasitic microbes maternally transferred; *Wolbachia* is extensively explored. *Wolbachia* is an intracellular Gram-negative bacterium that infects many arthropod insect species in nature. Until recently, its infection was considered parasitic, because it leads to several reproductive abnormalities in its host. Some *Wolbachia* strains can reproductively modify their hosts. One type of modification, called cytoplasmic incompatibility (CI), occurs when *Wolbachia*-infected males mate with uninfected females or females infected with an incompatible strain of *Wolbachia*, resulting in early embryonic death of their offspring. Moreover, multiple *Wolbachia* strains have been shown to confer resistance to viral infections in their native hosts. For example, many native *Wolbachia* infections in *D*. *melanogaster* (wMel, wMelCS, and wMelPop) and *D. simulans* (wAu and wRi) have been shown to provide viral protection to their hosts [98,99,100,101,102,103,104,105,106]. Additionally, the *Wolbachia* strain wPip has also been shown to increase the resistance to West Nile virus in its native mosquito host, *Culex quinquefasciatus*. Considering the capabilities of Wolbachia, it provides a promising tool for controlling disease vectors, thereby reducing virus transmission. Recent studies have shown that, when *Aedes aegypti* mosquitoes are transinfected with various *Wolbachia* strains, both CI and the resistance to viral infection are also conferred to their novel host. *A*. *aegypti* mosquitoes infected with the wMel and wAlbB strains of *Wolbachia* have been released in field trials in different countries such as Australia, Malaysia, and Indonesia as a strategy for controlling dengue [107,108,109,110,111]. New mosquito lines infected with other *Wolbachia* strains such as wMelCS, wRi, and wPip are currently under investigation for their effectiveness in disease control and as candidates for release in field trials [111]. 

Symbionts remain in harmony inside the arthropod vector. They establish homeostasis in host tissues by utilising either their molecules or different host-derived factors. Symbiotic microbes use various strategies and mechanisms to prevent the activation of the hostile immune system of the hosts. Whereas hosts also adjust their immune responses to support beneficial symbiosis and keep a check on symbiont growth. The gut is the crucial site for pathogen entry, and its condition decides the fate of the pathogen, i.e., its colonisation and survival. The cells of the host epithelium release a constitutive rush of antimicrobial peptides (AMPs) via activation of the immune deficiency (Imd) pathway and a high level of reactive oxygen species (ROS) to manage microbial outgrowth in the gut [112]. However, multiple negative regulatory elements of the Imd pathways have been reported to avoid damaging effects on native gut microbes. In *Drosophila*, pathogen recognition proteins (PGRPs), PGRP-LB, and PGRP-SC could scavenge peptidoglycan (immunostimulatory) and enable host tolerance to commensal microbes in the gut. Another regulator of the Imd pathway PGRP-LC-interacting inhibitor of Imd signalling (PIMS) could translocate the PGRP-LC receptor (activator of the Imd pathway) from the cell membrane to the intracellular compartment. The translocation of PGRP-LC from the plasma membrane inhibits Imd signalling to commensal bacteria [113]. The mosquito gut microbiome modulates the expression of C-type lectins (mosGCTLs) and coats bacterial surface ligands (polysaccharides) with mosGCTLs. It not only evades interactions between AMPs and the bacterial surface, but it also hides bacterial ligands from the pattern recognition receptors (PRRs) present in the gut epithelium [114]. The dual oxidase (DUOX)-dependent ROS production, a bona fide defence mechanism, was demonstrated in *Drosophila* gut epithelia for the controlled maintenance of a nutritional microbe: yeast [115].

The effective expression and regulation of ROS synthesising the Duox enzyme maintain the homeostatic condition and a healthy gut–microbiota interaction. In *Aedes aegypti* and phlebotomine sandflies, ROS was found to maintain the composition and homeostasis in the gut microbiome. However, limiting ROS production resulted in dysbiosis in *Aedes aegypti* [116,117]. The transinfected *Wolbachia* strain, wAlbB, demonstrated an increased ROS level followed by the upregulation of different antioxidant genes in *A. aegypti*. The antioxidant-mediated regulatory feedback prevents cell damage and maintains a persistent *Wolbachia* infection [118]. On the contrary, a *Wolbachia* natural infection neither upregulates nor suppresses the AMP-mediated immune response in insect hosts [119,120]. Such an observation could plausibly be because, being located within vesicles, *Wolbachia* hides from the host immune system and, therefore, does not induce AMP gene expression, or the host favours the maintenance of the bacteria by shutting down the AMP immune response [121].

Besides immune system modulation, bounding a physical barrier around microbes in vectors also limits the microbial fauna and contributes to homeostasis by evading the adverse host effects. An immunomodulatory peroxidase (IMPer)/Duox system uncovered in the *Anopheles gambiae* midgut epithelium forms a dynamic and transient di-tyrosine network upon blood feeding. This protein network decreases the flow of immune elicitors and its interaction to PRR present on the midgut cell membrane. It promotes commensal bacteria proliferation and protects the gut microbiota; instead, it makes *A. gambiae* susceptible to *Plasmodium* infection [122]. *Wolbachia* infection corroborates the above described by being restricted to cytoplasmic vesicles near the cell membrane [123]. The membrane of these vesicles is derived from the host that allows *Wolbachia* to hide from the host immune system. In new infestations, it triggers immune activation, whereas coevolved symbiosis involves stealthy growth inside the host via the suppression and interference of host immune responses. It requires the maintenance of redox homeostasis by balancing redox activation with the induced expression of antioxidants [124]. Therefore, conclusively, both microbial fauna and arthropod hosts tune up at the microbe–host interface for effective symbiosis.

### 4.2. Insect as a Carrier of Plant and Mammalian Pathogens

Besides beneficial microbes, insects also shelter microbes pathogenic to mammals or plants. They encompass over 130 arboviruses that cover the *Flaviviridae*, *Reoviridae*, *Togaviridae*, *Orthomyxoviridae,* and *Bunyaviridae* families. Insect-vectored viruses pose serious public health issues to humans, such as the Chikungunya virus, Dengue virus, West Nile virus, Japanese encephalitis, Yellow fever, and Zika virus. Insects acquire such pathogens while feeding on infected hosts and transmit to healthy hosts in subsequent feedings. These prolific insect vectors crave vertebrate blood to support egg development [125]. These vectors have expanded their geographic range due to global transport, mushrooming urbanisation, and climate change. As we lack efficacious vaccines against vector-borne pathogens, insecticides are a mainstay. However, insecticide-based vector control is in jeopardy due to the emergence of the resistance in the natural population [126]. It diverted substantial effort to procuring genetic information, parallelly unravelling insect biology and its interactions with pathogens. Its prime focus is gathering evidence on different aspects of vector physiology and vector competence (an insect’s ability to transfer pathogens) to stop transmissions, an effective alternative to massive insecticide usage [127]. It uncovers an unprecedented research area involving tripartite interactions among arthropod vectors–symbionts–arboviruses.

### 4.3. Tripartite Interaction of Symbionts–Arthropod-Borne Pathogens–Insect Vectors

The investigation of endosymbionts that featured their role in thwarting host vector competence is prime. Indeed, microbiota determined the vector susceptibility to arboviruses by modulating immune responses. The antibiotic treatment seemed more beneficial for DENV infections in mosquitoes than mock-treated mosquitoes [89]. In both *Anopheles stephensi* and *A. albimanus*, a *Plasmodium* vector, mosquitoes reared aseptically showed enhanced susceptibility to *Plasmodium* infection. Again, though, the susceptibility to pathogen infections was lowered compared to the normal by mere cofeeding *Plasmodium* and mosquitoes’ bacteria. The large-scale gene profiling of mosquitoes reared in septic and aseptic conditions featured a significant induction of immune genes and anti-*Plasmodium* factors, possibly by the microbes of the host. The gut microbes are now known to induce a basal level of the host’s antiviral immunity [128,129,130]. Similarly, depriving tsetse flies of *Wigglesworthia* (an obligate, commensal microbe of a tsetse fly) made flies highly susceptible to *Trypanosoma* infection [131].

Based on the interaction of microbes with vector-borne parasites, the microbiota can impact the vector competence of insect hosts by both direct and indirect means. Under the direct means, microbes hold a direct influence on the parasite through some metabolite secretions. For example, *Chromobacterium* secrets an aminopeptidase that directly degrades the envelope protein of DENV [132]. In *A. aegypti* caught from the field, *Serratia odorifera*, a commensal bacterium, was discovered to promote DENV-2 infection through some polypeptide secretion. The inoculation of *A. aegypti* with *S. odorifera* was found to increase CHIKV infection [133]. Alternatively, microbes can halt pathogen growth indirectly by modulating the host’s physical status and immune system. The symbionts can induce the host immune system and antiviral mechanisms such as AMP production, ROS burst, and Imd and Toll signalling pathways. A study conducted in the mosquito population from Zambia identified *Enterobacter* bacterium. The *Enterobacter*-induced ROS showed anti-Plasmodium effects [134]. Intriguingly, gut microbes play a vital role in synthesising and maintaining the peritrophic matrix (PM), preventing pathogen invasion in insect guts after a blood meal. Dysbiosis or the loss of gut commensal bacteria severely affects the PM and, ultimately, pathogen colonisation in insect vector gut epithelium [135,136,137,138]. PGRP-LB exhibits a dual role in tsetse flies.

Conversely, it negatively modulates the Imd pathway and protects *Wigglesworthia* (a mutualistic symbiont). On the other hand, a higher expression of PGRP-LB curtails the establishment of *Trypanosoma*. The microbial fauna seems to prime the host immune system and enhance the immune response to subsequent parasite challenges. *Wigglesworthia* does not directly influence the tsetse fly immunity to *Trypanosoma*, although its presence during the immature larval stages in adult flies marks the proper development and function of the immune system in developing larvae [33,139]. Correspondingly, an intimate association was found between beneficial gut microbes and haemocytes. In *A. gambiae*, an invasion by *Plasmodium* in the midgut increases the abundance of granulocytes in the insect hemocoel and enhances the immunity to bacteria while reducing viral reinfection [140]. Similar manifestations of immune priming of the host were observed in some *Wolbachia* infections. wMelPop *Wolbachia* trans-infection in A. aegypti was shown to upregulate immune genes, which may contribute to resistance to viral infection, although it is clear that other factors also contribute [141]. Thus, both direct and indirect means of pathogen blocking can significantly impact the vectorial capacity of the insect host. 

#### Wolbachia: A Panoply of Tactics for Vector-Borne Disease Control

The unravelling of *Wolbachia’s* role in interfering with viral replication in *Aedes aegypti* prompted inquisitiveness about the mystery of the mechanism employed by *Wolbachia* in virus blocking. It also pronounced *Wolbachia* as a potential biocontrol agent. Besides multiple *Wolbachia* strains, wMel and wAlbB could effectively block viral transmission [111]. The mechanism of virus blocking is enigmatic [142]. It might be achieved by several contributing factors that include (a) immune activation by the ROS-dependent Toll pathway (b) using host microRNAs and the (c) density of *Wolbachia* in crucial tissues, which also impact the extent of virus blocking [118,143,144,145,146]. The *Wolbachia* population competes with viruses for host resources (like cholesterol) and other molecules. The artificial introduction of *Wolbachia* strains such as wMel or wMelPop triggered cytoplasmic incompatibility in wild *A. aegypti* populations, leading to pathogen interference phenotypes by modulating the immune system and metabolic pathways. Cytoplasmic incompatibility (CI) is unrelated to pathogen interference. CI allows for *Wolbachia* to increase in frequency in the population. When a large proportion of mosquitoes are infected with *Wolbachia*, this will result in a decreased transmission of pathogenic viruses such as DENV and ZIKV [144,147,148]. Viruses like DENV and WNV are heavily dependent on cellular lipids, whereas the lipidome is perturbed upon *Wolbachia* infection [148,149]. The lipid profile of acyl-carnitines (a class of lipid) was prominently altered [150,151]. Acyl-carnitines are important intermediates involved in FA-CoA transport to the mitochondria further used for β-oxidation and ATP production. The viral infection elevates the acyl-carnitine levels in *Aedes aegypti* and disrupts signalling in mitochondrial functions, leading to diversions in cellular energy production [151]. In contrast, *Wolbachia* decreases the acyl-carnitine level and promotes FA-CoA catabolism. *Wolbachia* infection induces changes but does not perturb the cell homeostasis [152]. Another mechanism that *Wolbachia* utilises for pathogen blocking involves the downregulation of the activity of the insulin receptor kinase. The decrease of insulin receptors inhibits insulin signalling, reducing virus replication [153]. Insulin signalling is linked to acyl-carnitine. Therefore, the building up of acyl-carnitine upon viral infection could impair insulin signalling [154]. *Wolbachia* also modulates the homeostasis of the lipid and cholesterol to inflict pathogen blocking [155].

Recent studies showed that wMel *A. aegypti* mosquitoes could be successfully deployed at a large scale to control dengue in different countries like Australia [156], Indonesia [157], and Brazil [158]. Furthermore, agricultural pests such as aphids, planthoppers, and whiteflies cause severe damage to crops through feeding or by transmitting plant viruses [159,160,161]. A recent study suggested that the introduction of *Wolbachia* strain wStri into planthoppers, *Nilaparvata lugens,* inhibited infection and viral transmission in rice plants, thereby opening up new avenues in the development of Wolbachia-based control strategies against agricultural pests [162].

## 5. The Extended Microbial Contribution in Insect–Microbiome Interaction: A Quantum Leap

The microbiota that colonise insects contribute significantly to alleviating dietary challenges and maintaining homeostasis in the gut by facilitating host immunities. Besides, they mediate a wide array of ecologically important traits in insects and structure their functions, ecology, and evolution. For example, *Candidatus Westeberhardia cardiocondylae*, a gut-associated symbiont of *Cardiocondyla obscurior,* contributes to host cuticle formation and promotes an invasive lifestyle. *Westeberhardia* resides in ovarian nurse cells and is vertically transmitted [27]. In *Daceton armigerum*, 16S rRNA and 18S rRNA analyses of the microbial communities revealed different ecological and evolutionary factors shaping the host microbial communities [163]. A high-throughput sequencing technology of *Temnothorax nylanderi* ants identified the impacts of the environment and season on the diversity of the abdominal microbiome of ants rather than their caste [164]. Therefore, we will briefly address the microbiome’s role in determining host behaviour, physiology, and evolution.

### 5.1. Vitamin B Provisioning in Insect Nutrition

Insect genomics has demonstrated insects’ inability to synthesise B vitamins de novo. It also offered the insight of microbial contributions to supplement B vitamins to insects feeding on a diet deficient in B vitamins (vertebrate blood and plant sap). The axenic insects show depressed performances and require a regular supply of seven B vitamins [165,166]. The genomics analysis of *Wolbachia*, a symbiont in *Cimex lecticularis*, revealed a complete biosynthetic pathway for synthesising B vitamins (B2 and B7) [21]. Similarly, *Baumannia*, a symbiont of sharpshooters (sap feeder), is genetically capable of synthesising several B vitamins [31]. In another sap-sucking insect, *Acyrthosiphon pisum*, similar B vitamin provisioning was observed by *Buchnera*, a bacteriocyte-localised bacterial symbiont [32]. The requirement of the same set of B vitamins in insects as mammals and the absence of symptoms to individual B vitamin deficiencies undervalue the importance of insect models in exploring the role of B vitamins in humans. However, the immense diversity in insect nutritional ecology, microbial mutualists, and advancement in genomics and analytical techniques offer a ray of hope in vitamin B nutrition.

### 5.2. Microbial Secondary Metabolite-Driven Insect Community Interactions

A complicated multipartite symbiosis encompasses insect communities. These community interactions are commanded by a wide array of secondary metabolite secretions by insect-associated microbes. These complex networks of interactions ultimately shape insect symbiosis. For example, the antennal gland in solitary wasps, i.e., beewolves, cultivate the bacterium *Streptomyces* in their antennal glands. This bacterium monoculture produces piericidin polyketide (an antifungal compound). It protects larvae and enhances their survival [167]. Another example is from entomopathogenic nematodes *Steinernema* and *Heterorhabditis* that prey on various insects using their bacterial symbionts *Xenorhabdus* and *Photorhabdus*. These nematode-associated bacterial symbionts are released in insect haemolymphs by juvenile infective nematodes. The bacteria grow inside the insect and kill it. This provides a nutrient-rich breeding ground to nematodes that feed on bacteria, and the dead insects remain until the next attack. *Xenorhabdus* and *Photorhabdus* produce various secondary metabolites to suppress insect immune responses and evade opportunistic microbes’ growth on insect cadavers [168]. Similarly, fungus-growing ants (Attine ants) grow “cultivar fungus” (*Leucoagaricus*) for food. *Escovopsis* parasitise the cultivar fungus. To defend a cultivar fungus, attine ants maintain *Pseudonocardia* (an obligate bacterium) in the crypts of the cuticle to defend the cultivar fungus. *Pseudonocardia* produces antifungal secondary metabolites to inhibit *Escovopsis* (a parasitic fungus). A particular trait in such multipartite symbiosis experiences various sources of selection, resolved by a trade-off. Its goal is to enhance the overall fitness of symbiosis by emphasising a particular molecular interaction [44,169]. 

### 5.3. Microbiome-Shaping Insect Behaviour

All insect groups harbour a broader microbiome in addition to endosymbionts. The key difference is in their mode of transmission. Endosymbionts are transferred via a maternal transmission with high fidelity. In comparison, the broader microbiome does not rely much on maternal transmission and is transmitted via environmental factors. Vertically transmitted symbionts inherited from mother to offspring persist as mutualists influencing the host fitness or reproductive manipulators [170]. Some of these reproductive manipulators induce cytoplasmic incompatibility or are sex ratio distorters that increase the ratio of female offspring in the infected population, thereby altering the dynamics of sexual selection [171,172]. The bacterial symbiont Wolbachia modulates insect reproductive behaviours via feminisation and male-killing [173]. Other symbionts such as Rickettsia and Siproplasma also influence the sex ratio in diverse arthropod hosts such as Drosophila [174], spiders [175], and mites [176]. Another reproductive behaviour manipulator, Cardinium, influences the oviposition choice of Encarsia pergandiella (parasitoid wasps). This behavioural manipulation is induced to increase infected daughters in the population [177]. The infection of the entomopathogenic fungi Cordyceps (the “Zombie fungus”) causes a loss of appetite in their host, losing their coordination. The insects infected by the zombie fungus attach to foliage and later die. The fungal-spouting body develops on the dead host, bursts at maturity, and showers infected spores on the area below it [178]. Entomophthora muscae (a parasite of a housefly) induces necrophilia in uninfected males in a bizarre situation. They get more attracted to dead, infected females than uninfected ones [179]. Viral infection by IIV-6/Cr IV manipulates the mating behaviour of infected male Gryllus texensis and alters their courtship singing pattern [180]. The bacterial symbiont composition of the host is also altered upon interspecies competition. Bacterial communities buffer behavioural changes upon biological invasions [181]. Behavioural manipulation is the most exciting phenomenon in insect–microbiome interactions [12].

### 5.4. Gut Microbiota Linking Insects’ Nervous System, Physiology, and Behaviour

Recently, the link between the gut microbiota and animal neurophysiology and its behaviour has gained an exponential thrust. The studies conducted on mammalian models are now extrapolated to insect systems. They have identified the same molecular mechanisms in insects as that of mammals. Insects—mainly social insects—are amenable models to study specific gut microbes in behavioural dysfunctions [6]. The gut microbiota secretes various neuroactive compounds to modulate brain functions. It has been suggested that an episode of microbial dysbiosis could lead to social dysfunctions, like schizophrenia and autism spectrum disorders (ASD) [182,183]. Insect intestine-associated microbiota contribute to its cognition, development, social interactions, and chemical communication. By altering the odorant profile of insects, gut microbes alter their behaviours, such as aggregation, mating, and foraging [184,185,186,187,188]. The profound influence of gut-associated microbes has been discovered in insects’ neurophysiological development of cognition, such as learning and memorisation. A parkin gene from *Drosophila* has been linked to Parkinson’s disease in humans [4]. The *Drosophila* model has been successfully utilised to study Alzheimer’s disease, and its symptoms were ameliorated in flies by a probiotic supplementation with *Lactobacillus* and *Bifidobacterium* strains [189].

Furthermore, researchers have established a link between the reduced expression of histone demethylase KDM5 genes (associated with ASD symptoms) and the alteration in the gut microbiota composition in a *Drosophila* model. These ASD symptoms can be rescued in flies by probiotic supplementations with *Lactobacillus* strains [190]. The future outlook should be disentangling the evolutionary origin of the gut microbiota–brain axis and finding suitable probiotics to cure cognitive and behavioural dysfunctions.

## 6. Insect Symbiosis: Implication and Outlook

Insects being the largest and most diversified group on Earth drives several significant roles in the ecosystem. They harbour an astonishing array of microbial communities. The overwhelming impact of microbes on insect functions, ecology, and evolution has gained immense attention recently. Their intimate associations with insect physiology, behaviours, and reproduction have enormously changed our perspective. Understanding the strategy deployed by microbes to manipulate insect hosts would unearth new bioactive molecules, having great potential in medicine [191]. As insects are severe agricultural and forest pests and carriers of vector-borne diseases, unravelling pest insects as holobionts will be of great potential in future IPMs. With *Wolbachia* being the leading one, paratransgenesis (engineering multiple blocking factors into one microbial species) is an impressive tool for vector-borne disease control [127]. Lately, the influence of insect gut microbiota on dsRNA treatment has also been observed. The synergistic effect of the microbiome on RNAi-mediated insect pest control has opened up a new avenue of research [192]. The insect gut microbiome can also serve as carriers for dsRNA delivery (SMR: symbiont-mediated RNAi), leading to the sustainable and species-specific delivery of RNA interference [193]. The elucidation of the gut microbiota–brain axis has established insects as potential models for decoding the role of microbes in neurological dysfunctions and their possible probiotic treatment [6]. Future studies on resident microbes within insects using advanced omics approaches such as metatranscriptomics, metaproteomics and metametabolomics will unravel the molecular exchanges underlying symbiosis in model and non-model insects and, thus, shed light on their eco-evolutionary implications in greater depth.

## Figures and Tables

**Table 1:** A glimpse of symbiotic relationships in insects.

Bacterial Symbionts	Insect Host	Niche Location within Host	Transmission Mode	Interaction Benefits	References
*Ishikawaella capsulate* (Obligate mutualist)	*Megacopta punctatissima* (Plataspid stinkbugs)	Extracellular midgut	Inheritable and transmitted through a capsule	Enhance pest status of the insect host. Microbe compensates for nutritional deficiency of host diet by supplying essential amino acids.	[16,17]
*Regiella insecticola* (Facultative commensal)	*Acyrthosiphon pisum* (Aphid)	Bacteriocytes, Haemolymph	Inheritable and transmitted via Transovarial	Influence host plant range; survival, and reproduction on clover of insect host.	[18]
*Wolbachia* sp. (Facultative parasite)	*Diabrotica virgifera*	Bacteriocytes, extracellularly scattered	Inheritable and transmitted via Transovarial	Silencing of maize (host plant) defence induction via insect host.	[19]
*Regiella insecticola* (Facultative commensal)	*Myzus persicae* (peach-potato aphid)	Bacteriocytes, Haemolymph	Inheritable and transmitted via Transovarial	Protection against parasitoids.	[20]
*Wolbachia* sp. (Facultative parasite)	*Cimex lectularius*	Bacteriocytes, extracellularly scattered	Inheritable and transmitted via Transovarial	Provisioning of B vitamins.	[21]
*Candidatus liberibacter Psyllaurous* (Facultative)	*Bactericera cockerelli* (Tomato psyllid)	Extracellular	Acquired during feeding and vectored by the insect host	Reduced expression of plant defensive gene in tomato probably for psyllid success.	[22,23]
*Wolbachia* sp. (Facultative parasite)	*Phyllonorycter blancardella* (Leaf mining moth)	Bacteriocytes, extracellularly scattered	Inheritable and transmitted via Transovarial	To increase host insect fitness, the maintenance of chlorophyll and nutrient-rich “green island” (insect feeding site) in senescent leaves of the host plant.	[24]
*Buchnera* spp. (Obligate mutualists)	*Bemisia tabaci* (Whitefly)	Mycetocytes	Inheritable and transmitted via Transovarial	Produces GroEL chaperone protein that binds to plant viruses and makes virus transmission efficient.	[25]
*Hamiltonella* (Facultative Commensal)	*Bemisia tabaci* (Whitefly)	Sheath Cells, Secondary Myocetocytes, Haemolymph	Acquired and Inheritable; Horizontal and Maternal	GroEL protein produced by *Hamiltonella* facilitates transmission of tomato yellow leaf curl virus vectored by whitefly.	[26]
*Candidatus Westeberhardia cardiocondylae*	*Cardiocondyla obscurior (Invasive ant)*	Gut-associated bacteriomes	Transmitted to late-stage oocytes; Vertical transmission	Contributes to cuticle formation and is responsible for host invasive success.	[27]
*Hamiltonella* (Facultative Commensal)	*Acyrthosiphon pisum* (Pea aphid)	Sheath Cells, Secondary Myocetocytes, Haemolymph	Acquired and Inheritable; Horizontal and Maternal	It confers resistance to host insects from a parasitoid attack.	[28]
*Regiella insecticola*(Facultative commensal)	*Acyrthosiphon pisum*	Bacteriocytes, Haemolymph	Inheritable and transmitted via Transovarial	Resistance to host insect from fungal pathogens	[29]
*Burkholderia* sp.	*Riptortus pedestris*	Crypts at posterior midgut region	Acquired from environment and undergo horizontal transmission	Symbiont-mediated fenitrothion (insecticide) resistance to insect host	[30]
*Baumannia cicadellinicola* (obligate mutualist)	Sharpshooters	Bacteriocytes	Inheritable and transmitted via Transovarial	*Baumannia* contributes several B vitamins to its host insect.	[31]
*Buchnera* spp. (Obligate mutualists)	*Acyrthosiphon pisum*	Bacteriocytes	Inheritable and transmitted via Transovarial	*Buchnera* contributes several B vitamins to its host insect.	[32]
*Wigglesworthia glossinidia*(Obligate mutualist)	Tsetse flies	Bacteriocytes	Inheritable and transmitted via Transovarial	*Wigglesworthia* presence during the development of larval stages is vital for Tsetse flies’ immune system development and function.	[33]
*Sodalis glossinidius* (Secondary facultative)	Tsetse flies	Numerous tissues	Both inheritable and acquired; Transmitted via milk gland, mating and transovarial	*Sodalis* impacts tsetse flies vector competence and longevity	[34]
*Serratia symbiotica* (Facultative symbiont)	Aphids	NA	Acquired from the environment; Horizontal transmission	In the *Lachninae* subfamily, *Serratia* supplements *Buchnera aphidicola* ability of tryptophan biosynthesis. In *Acyrthosiphon pisum, S. symbiotica* is involved in heat stress tolerance and parasitoid resistance to host insect.	[35]
*Serratia marcescens* (Facultative symbiont)	hematophagous insects	midgut	Adhere to eggs surface, colonize oviposition site	*Serratia marcescens* have an anti-Plasmodium function in *Anopheles* mosquito midgut	[36]
**Fungal Symbionts**	**Insect Host**	**Niche location within the host**	**Transmission mode**	**Interaction Benefits**	**References**
*Grosmannia clavigera* (Obligate mutualist)	*Dendroctonus ponderosae* (Bark beetle)	Mycangia, exoskeleton	Acquire spores in the pupal chamber just before emergence	Increased success of host insect on jack pines (host plant) reduces food quality for interspecific competitors	[37]
				Oxygenated monoterpenes produced by microbial activity is used as host (beetle) location cues by parasitoids.	[38]
				*Grosmannia clavigera* can detoxify oleoresin terpenoids (conifer-defence chemicals) and utilize them as carbon sources. It allows host insects to tolerate terpenoids and grow successfully on pine hosts	[39]
*Raffaelea lauricola* (obligate mutualist)	*Xyleborus glabratus* (Redbay ambrosia beetle)	Mycangia, exoskeleton	Larvae and adults feed on the conidia	Volatile cues from fungal symbionts may function as a mechanism to locate established fungal gardens of conspecific beetles (suitable microhabitat) but also as an orientation cue within a gallery	[40]

## Data Availability

The data presented in this study are available in this article only.

## Abbreviations

PM: peritrophic matrix; PCR: polymerase chain reaction; Imd: immune deficiency (Imd) pathway; Duox-ROS: dual oxidase-reactive oxygen species; DAP-PGN: Diaminopimelic acid (DAP)-type peptidoglycan (PGN); PGRP: PGN recognition peptide; AMPs: antimicrobial peptides; Dome: domeless; Hop: hopscotch; Upd: unpaired; TRAF3: tumour necrosis factor receptor (TNFR)-associated factor 3; JAK-STAT: Janus kinases (JAKs), signal transducer and activator of transcription proteins (STATs) and receptors; CI: cytoplasmic incompatibility; ROS: reactive oxygen species; PGRPs: pathogen recognition proteins; PIMS: PGRP-LC-interacting inhibitor of Imd signalling; mosGCTLs: mosquito galactose-specific C-type lectins; PRRs: pattern recognition receptors; IMPer: immunomodulatory peroxidase; DENV: Dengue virus; CHIKV: Chikungunya virus; WNV: West Nile virus; ASD: autism spectrum disorders; and SMR: symbiont-mediated RNAi.
